# Identifying Differentially Expressed Genes in Pollen from Self-Incompatible “Wuzishatangju” and Self-Compatible “Shatangju” Mandarins

**DOI:** 10.3390/ijms14048538

**Published:** 2013-04-17

**Authors:** Hongxia Miao, Zixing Ye, Jaime A. Teixeira da Silva, Yonghua Qin, Guibing Hu

**Affiliations:** 1 State Key Laboratory for Conservation and Utilization of Subtropical Agro-bioresources, College of Horticulture, South China Agricultural University, Guangzhou 510642, China; E-Mail: guibing@scau.edu.cn; 2 Key Laboratory of Biology and Genetic Improvement of Horticultural Crops-South China of Ministry of Agriculture, College of Horticulture, South China Agricultural University, Guangzhou 510642, China; E-Mail: qinyh@scau.edu.cn; 3 Faculty of Agriculture and Graduate School of Agriculture, Kagawa University, Ikenobe, Kagawa 761-0795, Japan; E-Mail: jaimetex@yahoo.com

**Keywords:** *Citrus reticulata* Blanco, self-incompatibility (SI), suppression subtractive hybridization (SSH), pollen, expression analysis

## Abstract

Self-incompatibility (SI) is one of the important factors that can result in seedless fruit in *Citrus*. However, the molecular mechanism of SI in *Citrus* is not yet clear. In this study, two suppression subtractive hybridization (SSH) libraries (forward, F and reverse, R) were constructed to isolate differentially expressed genes in pollen from “Wuzishatangju” (SI) and “Shatangju” (self-compatibility, SC) mandarins. Four hundred and sixty-eight differentially expressed cDNA clones from 2077 positive clones were sequenced and identified. Differentially expressed ESTs are possibly involved in the SI reaction of “Wuzishatangju” by regulating pollen development, kinase activity, ubiquitin pathway, pollen-pistil interaction, and calcium ion binding. Twenty five SI candidate genes were obtained, six of which displayed specific expression patterns in various organs and stages after self- and cross-pollination. The expression level of the *F-box* gene (H304) and S1 (F78) in the pollen of “Wuzishatangju” was 5-fold higher than that in “Shatangju” pollen. The *F-box* gene, S1, UBE2, UBE3, RNaseHII, and PCP were obviously up-regulated in pistils at 3 d after self-pollination of “Wuzishatangju”, approximately 3-, 2-, 10-, 5-, 5-, and 2-fold higher, respectively than that at the same stage after cross-pollination of “Wuzishatangju” × “Shatangju” pistils. The potential involvement of these genes in the pollen SI reaction of “Wuzishatangju” is discussed.

## 1. Introduction

Self-incompatibility (SI) is a widespread mechanism in angiosperms which allows the pistil of a flower to reject self (genetically related) pollen, but to accept non-self (genetically unrelated) pollen for fertilization [1]. SI systems are categorized into sporophytic self-incompatibility (SSI) and gemetophytic self-incompatibility (GSI) according to the genetic control of pollen behavior. In the SSI system of Brassicaceae, growth of the pollen tube arrests at the surface of the stigma and a kinase-mediated signaling cascade is involved in the SI reaction [2]. In the GSI system of Rosaceae, Solanaceae, and Plantaginaceae, the arrest of growth of incompatible pollen tubes is in styles and its SI reaction is genetically controlled by style-expressed *S-RNase* (*S* locus-encoded Ribonuclease) genes [3] and pollen-expressed *SFB* (*S-haplotype-specific F-box*)/*SLF* (*S-locus F-box*) genes [4–6]. To date, style-expressed *S-RNase* genes have been isolated from the Solanaceae [7], Rosaceae [8], and Plantaginaceae [9]. However, pollen-expressed *S* gene remains a myth. In 2000, a cDNA fragment of the pollen *S* gene was first identified from *Nicotiana alata* and it was primarily expressed during pollen development [10]. Then, *AhSLF-S_2_* (*Antirrhinum hispanicum S*-locus F-box of *S_2_*-haplotype), a candidate pollen *S*-determinant gene [4], and *PiSLF_2_* (*S_2_*-allele of *Petunia inflata S*-locus F-box) [11] were obtained from *P. inflata*. Currently, great progress has been made in cloning, expression and functional analysis of pollen-expressed *SFB/SLF* genes from plum (*Prunus mume; SLF_1_*, *SLF_2_*, *SLF_3_*) [12], almond (*P. dulcis; SFBa*, *SFBc*) [5], Japanese apricot (*P. mume; SFB_f_*) [13], European apricot (*P. armeniaca; SFBc*) [14], sweet cherry (*P. avium; SFB_3_*, *SFB_9_*) [15], sour cherry (*P. cerasus; SFB_13_*_′_) [16], apple (*Malus* × *domestica; SLF_1_*, *SLF_2_*) [17], petunia (*P. inflata; SLF_2_*, *SLF_b_-S_2_*) [11,18], and Japanese pear (*Pyrus pyrifolia; PpSFBB*) [19].

*Citrus* belongs to the GSI system [20], and the study of SI in *Citrus* species is relevant not just because of the commercial impact (seedless fruit) it could have, but also for the understanding of SI systems evolution. GSI of *Citrus* is different from the GSI of model plants, and cytological analysis showed that the site of self-pollen tube inhibition was in the upper styles of “Commune” clementine (*C. clementina* Hort. ex Tan) [21], the lower one-third of styles of 29 *Citrus* cultivars [22], and the ovaries of “Guanxi” pomelo, “Duwei” pomelo (*C. grandis*) [23] and “Wuzishatangju” mandarin (*C. reticulata* Blanco) [24]. To date, style-expressed *RNase* activity genes have been obtained from Calamondin (*C. reticulata* var. *austera* × *Fortunella* sp.) [25], “Hirado Buntan” (*C. grandis* Osbeck) [26], “Zigui shatian” pummelo (*C. grandis* Osbeck) [27] and “Wuzishatangju” (*C. reticulata* Blanco) [28]. Recently, a full-length cDNA sequence of the *ubiquitin-activating enzyme E1* (*UBE1*) gene was obtained from the pistils of “Wuzishatangju” (SI) and “Shatangju” (self-compatibility, SC) mandarins, in which five amino acids in cDNA and DNA sequences of *UBE1* between “Wuzishatangju” and “Shatangju” differed, and in which the expression level of the *UBE1* gene in “Shatangju” anthers was approximately 10-fold higher than that in the anthers of “Wuzishatangju” (SI) [29]. Compared to “Wuzishatangju”, three amino acids were substituted in the cDNA of the *S_1_self-incompatibility locus-linked pollen 3.15* gene (*S_1–3.15_*) from “Shatangju” (SC). The expression level of the *S_1–3.15_* gene in the ovaries of “Shatangju” was approximately 60-fold higher than that in the ovaries of “Wuzishatangju” [30]. Moreover, pistil-expressed SI-related genes such as Ca^2+^-binding protein, senescence-associated cysteine protease, and C2-domain containing protein, were isolated from SI “Wuzishatangju” mandarin [31,32]. However, differentially expressed pollen *S* genes or other pollen SI-related factors in “Wuzishatangju” (SI) and “Shatangju” (SC) mandarins are still unknown.

“Wuzishatangju” (*C. reticulata* Blanco) (seedless, very tasty and easy-to-peel), derived from a bud mutation of a seedy cultivar “Shatangju”, is one of the most popular mandarin cultivars in China. Our previous cytological studies showed that the seedlessness of “Wuzishatangju” results from GSI [24]. To explore the molecular mechanism of SI in “Wuzishatangju”, two suppression subtractive hybridization (SSH) libraries were constructed to isolate pollen SI candidate genes using mature pollen of SI “Wuzishatangju” and SC “Shatangju”. Expression characteristics of all SI candidate genes were analyzed using semi-quantitative RT-PCR (SqRT-PCR) and quantitative real-time PCR (qPCR). The aim of this study was to identify differentially expressed genes in pollen from self-incompatible “Wuzishatangju” and self-compatible “Shatangju” mandarins and to discuss the possible roles of the identified candidate genes in the SI response of “Wuzishatangju”.

## 2. Results

### 2.1. Identification of Gene Fragments from Two SSH Libraries

Two SSH libraries were constructed to isolate differentially expressed genes from mature pollen of “Wuzishatangju” and “Shatangju” mandarins. According to the results from colony-PCR, a total of 2077 positive colonies (1050 from the forward SSH library (F) and 1027 from the reverse SSH library (R)) were obtained and the size of inserted cDNA fragments ranged from 300 to 1000 bp, although most were 400–700 bp (Figure S1). Based on reverse northern analysis, 230 clones from the F library were up-regulated in “Wuzishatangju” but down-regulated in “Shatangju” and 238 clones from the R library were up-regulated in “Shatangju” but down-regulated in “Wuzishatangju”. Up-regulated clones were identified using the DIG-labeled cDNA from “Shatangju” and “Wuzishatangju” (Figure S2).

### 2.2. General Statistics of Two SSH Libraries

**Figure 1:**
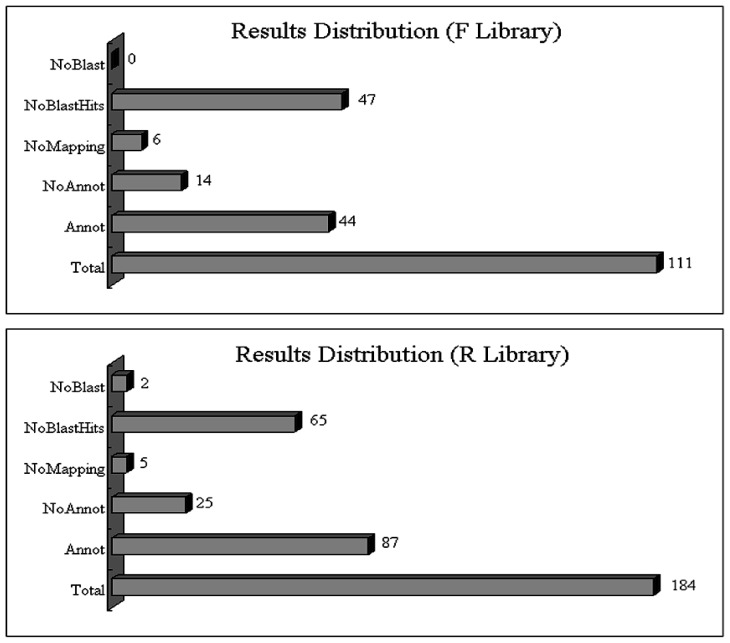
Gene distribution from two suppression subtractive hybridization (SSH) libraries. In the F library, self-incompatibility (SI) “Wuzishatangju” pollen was used as the “tester”, and self-compatibility (SC) “Shatangju” as the “driver”. In the R library, SC “Shatangju” pollen was used as the “tester”, and SI “Wuzishatangju” as the “driver”. The Arabic numerals represent the number of unique sequences at each step of the annotation process. NoBlast, no blast result; NoAnnot, no annotation; Annot, annotation. Data analysis and visualization of results were performed by the Blast2GO software.

The 468 differentially expressed clones (230 from the F library and 238 from the R library) were sequenced (BGI, Shenzhen, China). After removing repeat sequences, 111 and 184 unique sequences were obtained from the F and R library, respectively. All these sequences (295) were compared with an available database to find similarities with known sequences. Dynamic translation (Blastx) was carried out and only matched sequences with an E-value lower than 10^−3^ were considered to be homologous sequences while sequences with an *E*-value higher than 10^−3^ were labeled as undescribed. Homologous sequences accounted for 59.6% of the sequences in the F library and for 67.4% in the R library (Table S1). Among the homologous sequences, 14 from the F library (14/64, 21.8%) and 25 from the R library (25/117, 21.4%) lacked annotation. Additionally, there were a total of 11 (six from the F library and five from the R library) no-mapping sequences. All of these data are summarized in Figure 1.

### 2.3. Gene Ontology Analysis

**Figure 2:**
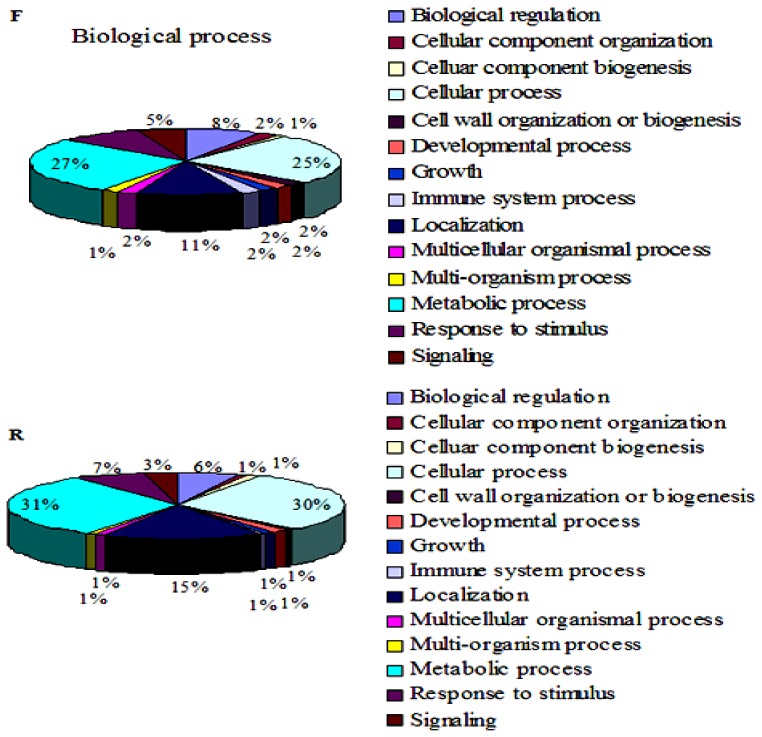
Classification of the unique sequence from the SSH according to Gene Ontology criteria. Gene Ontology analysis was carried out on the transcripts isolated from the two libraries. The graph, which combined biological processes, was made based on ontology level 2.

Gene ontology analysis was carried out using the blast2GO program and three ontological categories *i.e.*, biological process (Figure 2 and Table S2), molecular function and cellular components were obtained (Figure S3). In the category of “biological process”, the most frequent process was the metabolic process (27% in the F library and 31% in the R library), followed by the cellular process (25% in the F library and 30% in the R library) and localization (11% in the F library and 15% in the R library). For the “molecular function”, catalytic activity was the most frequent activity (53% in the F library and 49% in the R library), followed by binding protein (30% in the F library and 27% in the R library). Significant differences were observed in electron carrier activity (none in the F library and 1% in the R library) and transcription regulator activity categories (11% in the F library and 19% in the R library). For the “cellular component”, the most frequent activity was cell (49% in the F library and 50% in the R library), followed by organelle (39% in the F library and 37% in the R library). The macromolecular complex was different (5% in the F library and 10% in the R library) between the F library and the R library. According to the bioinformatics analysis, differentially expressed ESTs are possibly involved in the pollen SI reaction of “Wuzishatangju” through the regulation of pollen development, kinase activity, the ubiquitin pathway, the pollen-pistil interaction, sexual differentiation, and signal transduction (Table 1).

### 2.4. Expression Analyses of SI Candidate Genes by SqRT-PCR

**Figure 3:**
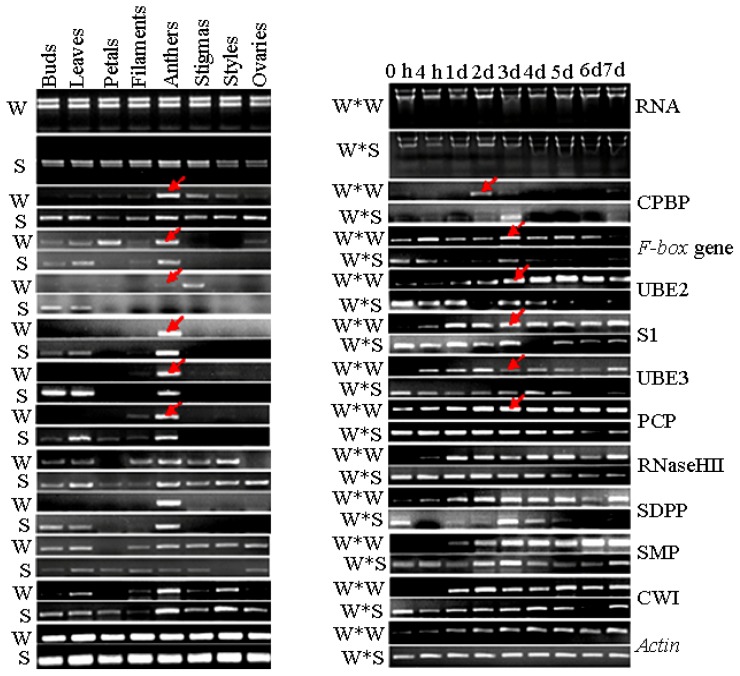
Expression analysis of 10 pollen SI-related genes in different organs of “Wuzishatangju” and “Shatangju” mandarins using SqRT-PCR. CPBP, Calcium-dependent phospholipid binding protein; UBE2, Ubiquitin-conjugating enzyme E2; S1-protein, self-incompatibility S1 family protein; UBE3, Ubiquitin-protein ligase E3; PCP, Pollen coat-like protein; RNase HII, Ribonuclease HII family protein; SDPP, Sexual differentiation process protein; SMP, Seed maturation protein; CWI, Cell wall invertase. Data are based on 0.5 g of random samples collected from each organ.

Based on the SqRT-PCR analyses, 25 candidate genes (10 from the F library and 15 from the R library) were chosen for further analyses (Table S3). Among the 25 candidate genes, there are nine potential pollen-pistil/pollination process genes (Table S4). Results from SqRT-PCR showed that expression levels of the *ubiquitin-conjugating enzyme E2* gene (UBE2) and a *WD-40 repeat protein-like* gene (WD-40) in pollen were lower than those of the other organs of “Wuzishatangju”. The other 23 genes displayed a similar organ-specific and up-regulated pattern in the pollen of SI “Wuzishatangju”. After self-pollination of “Wuzishatangju” and cross-pollination of “Wuzishatangju” × “Shatangju”, 10 SI-related genes *i.e.*, the *F-box* gene (H304), UBE2 (H858), UBE3 (H556), S1 (F78), RNaseHII (F465), SDPP (F960), PCP (H102), CPBP (H115), SMP (F966), and CWI (F1019) showed a time-specific expression pattern (Figure 3). The expression level of CPBP (H115) in pistils at 2 d after self-pollination of “Wuzishatangju” was higher than 2 d after cross-pollination of “Wuzishatangju” × “Shatangju”. PCP (H102), the *F-box* gene (H304) and S1 (F78) were obviously up-regulated in pistils at 3 d after self-pollination of “Wuzishatangju”. The highest expression level of SMP (F966) was detected in pistils at 6 d after self-pollination of “Wuzishatangju” while the lowest expression level was observed in pistils at 6 d after cross-pollination of “Wuzishatangju” × “Shatangju” (Figure 3).

### 2.5. Expression Analyses of SI-Related Genes in Different Organs from “Wuzishatangju” and “Shatangju” Mandarins Using qPCR

**Figure 4:**
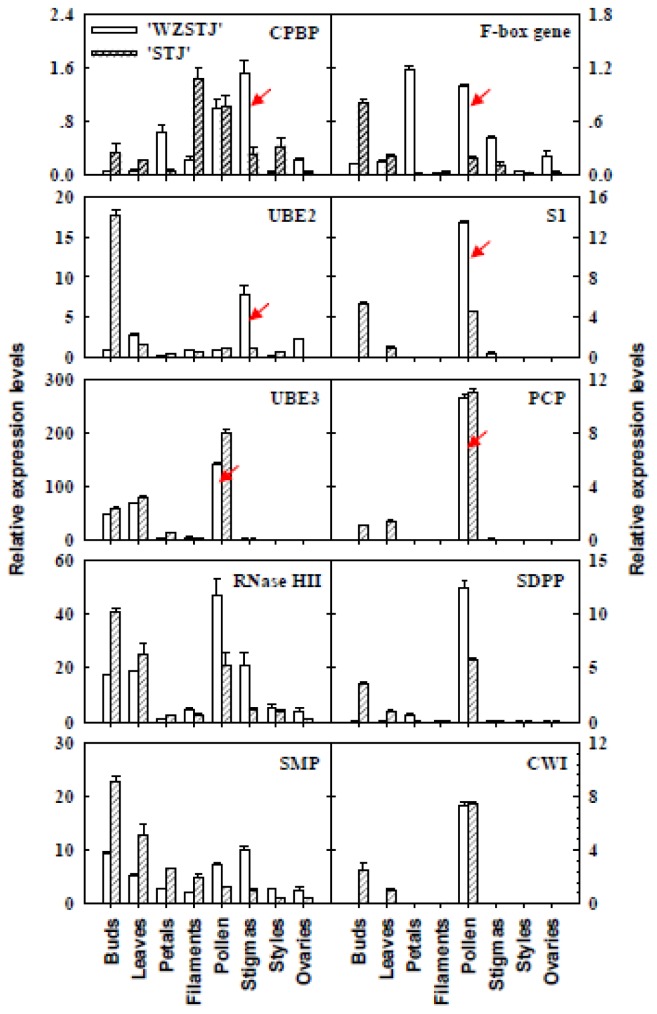
Expression analysis of 10 pollen SI-related genes in different organs of “Wuzishatangju” and “Shatangju” mandarins using qPCR. CPBP, Calcium-dependent phospholipid binding protein; UBE2, Ubiquitin-conjugating enzyme E2; S1-protein, self-incompatibility S1 family protein; UBE3, Ubiquitin-protein ligase E3; PCP, Pollen coat-like protein; RNase HII, Ribonuclease HII family protein; SDPP, Sexual differentiation process protein; SMP, Seed maturation protein; CWI, Cell wall invertase. The error bars represent SD of three replicates. Data are based on 0.5 g of random samples collected from each organ.

Organ-specific expression patterns of 10 SI-related genes in different organs from “Wuzishatangju” and “Shatangju” were further analyzed using qPCR (Figure 4). The expression level of the *F-box* gene in petals and pollen of “Wuzishatangju” was 3- and 2-fold higher, respectively than that in the same organ of “Shatangju”. A similar expression pattern of S1, RNase HII, and SDPP was detected in “Wuzishatangju” pollen, approximately 4-, 2-, and 3-fold higher, respectively than that in “Shatangju” pollen. The expression level of CPBP in the stigmas of “Wuzishatangju” was 4-fold higher than that in “Shatangju” stigmas. Compared to other organs, UBE3, PCP, and CWI were obviously up-regulated in pollen from “Wuzishatangju” and “Shatangju” mandarins.

### 2.6. Expression Analyses of SI-Related Genes in Different Stages of the Pistil after Self-Pollination of “Wuzishatangju” and Cross-Pollination of “Wuzishatangju” × “Shatangju” Using qPCR

**Figure 5:**
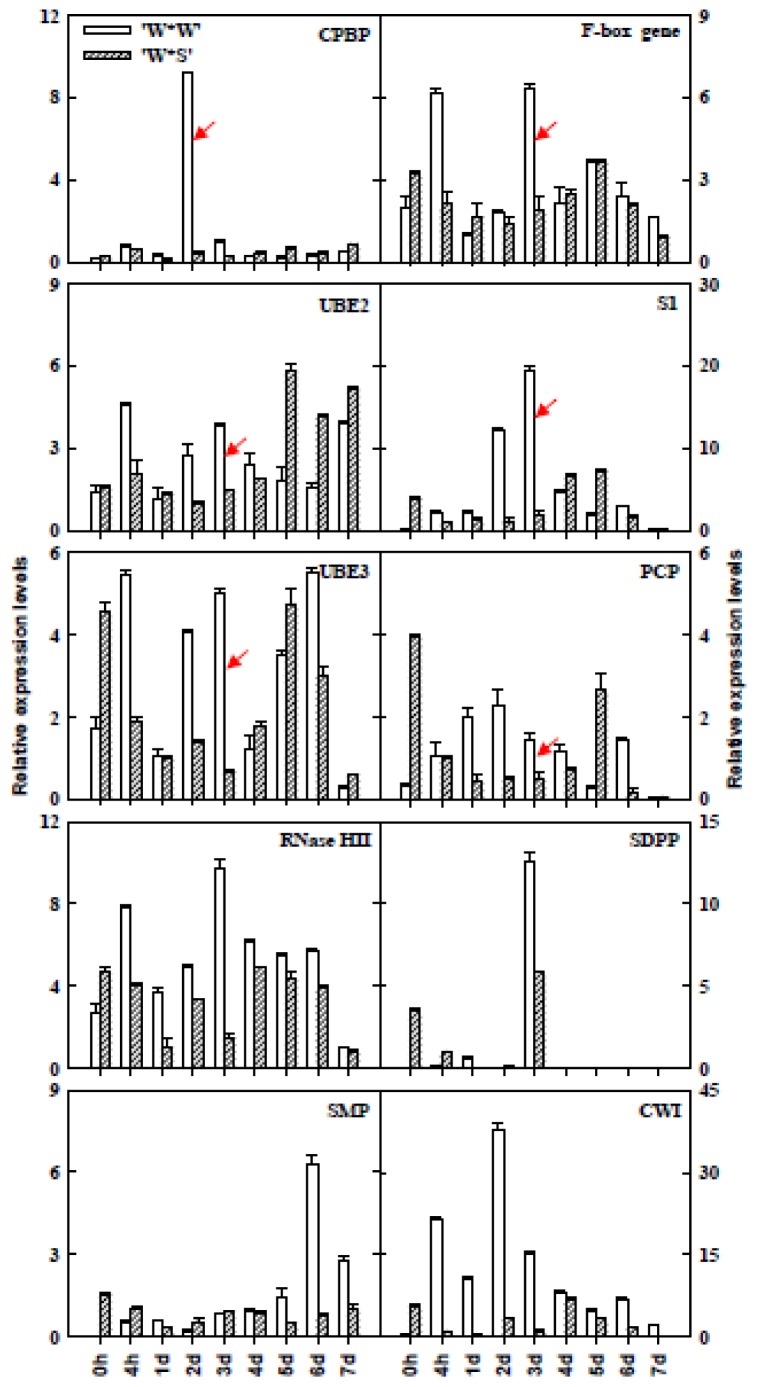
Expression analyses of 10 pollen SI-related genes in different stages of pistils after self-pollination of “Wuzishatangju” and cross-pollination of “Wuzishatangju” × “Shatangju” mandarins using qPCR. “W*W”, self-pollination of “Wuzishatangju” × “Wuzishatangju”; “W*Y”, cross-pollination of “Wuzishatangju” × “Shatangju”. CPBP, Calcium-dependent phospholipid binding protein; UBE2, Ubiquitin-conjugating enzyme E2; S1-protein, self-incompatibility S1 family protein; UBE3, Ubiquitin-protein ligase E3; PCP, Pollen coat-like protein; RNase HII, Ribonuclease HII family protein; SDPP, Sexual differentiation process protein; SMP, Seed maturation protein; CWI, Cell wall invertase. The error bars represent the SD of three replicates. Data are based on 0.5 g of random samples collected from each organ.

Time-specific expression patterns of 10 SI-related genes in different stages of pistils after self-pollination of “Wuzishatangju” and cross-pollination of “Wuzishatangju” × “Shatangju” were detected using qPCR (Figure 5). The highest expression levels of CPBP and CWI were detected in pistils at 2 d after self-pollination of “Wuzishatangju”, and were approximately 30- and 40-fold higher than 2 d after cross-pollination of “Wuzishatangju” × “Shatangju”, respectively. For self-pollinated pistils of “Wuzishatangju”, a decrease in the expression level of S1 was observed at 0 h, 4 h, 1 and 2 d, followed by an increase at 3, 4 and 5 d, and a decrease thereafter. Similar expression patterns of the *F-box* gene, UBE2, UBE3, PCP, RNase HII, and SDPP were detected in pistils at 3 d after self-pollination of “Wuzishatangju”, resulting in 3-, 2-, 5-, 5-, 4-, and 2-fold higher expression than 3 d after cross-pollination of “Wuzishatangju”× “Shatangju”, respectively. The expression level of SMP in pistils at 6 d after self-pollination of “Wuzishatangju” was 6-fold higher than 6 d after cross-pollination in pistils of “Wuzishatangju” × “Shatangju”.

## 3. Discussion

SSH technology is an effective method to screen different expression SI-related genes between the control (the driver) and the experimental transcriptome (the tester) [31–33]. Pollen-pistil interactions are an essential prelude to fertilization in angiosperms and determine SI/SC [34]. In the pistil’s SI response of “Wuzishatangju” mandarin, differentially expressed ESTs are possibly involved in the SI reaction through the regulation of the ubiquitin/26S proteasome pathway, the Ca^2+^-signaling pathway, receptor kinase, developmental processes, stimulus, or transcription [31]. Although pollen *S* genes or SI-related factors in different species have been reported, how these genes operate in *Citrus* is unknown. In this study, two SSH libraries were constructed to identify differentially expressed genes in pollen from “Wuzishatangju” (SI) and “Shatangju” (SC) mandarins. Several SI candidate genes involved in the kinase activity (*F-box* gene, H304, accession No. JK724779), pollen-pistil interaction (Self-incompatibility S1 family protein (S1), F78, accession No. JK724787), ubiquitin pathway (Ubiquitin-conjugating enzyme E2 (UBE2), H858, accession No. JK724783; Ubiquitin-protein ligase E3 (UBE3), H556, accession No. JK724782), calcium ion binding (Calcium-dependent phospholipid binding protein (CPBP), H115, accession No. 724776), and pollen development (Pollen coat-like protein (PCP), H102, accession No. 724776) (Table 1) were obtained.

A large number of *F-box* genes were found in model species [4,5,12,17,35]. *F-box* genes play an important role in the regulation of a complex set of developmental events during floral development [36] and the SI reaction [37]. Higher expression levels of the *CgF-box* were noted in the style, petals and anthers compared to low expression levels in the ovary and leaves of “Zigui shatian” pummelo (*C. grandis* Osbeck) [36]. In SI citrus clementina, a novel *F-box* gene showed a drastic up-regulation both in laser microdissected stylar canal cells and in self-pollinated whole styles with stigmas of “Comune”, concomitant with the arrest of pollen tube growth [38]. In the Rosaceae, specific expression of *SLF* (*S-*locus F-box) genes in pollen was responsible for SI in *P. dulcis*[5], *P. mume*[12], and *P. avium*[17]. In the present study, an *F-box* gene (H304) was obtained whose expression was obviously up-regulated in pollen of SI “Wuzishatangju” (Figure 4), a pattern which mimics the expression of *SLF/SFB* genes [12,17]. The highest expression of the *F-box* gene (H304) was found in pistils at 3 d after self-pollination of “Wuzishatangju” (Figure 5), which was consistent with our previous findings in which 3 d was the crucial period for a shift from a self-compatible to a self-incompatible state [28], implying that the *F-box* gene may be involved in the pollen development or SI response of “Wuzishatangju” mandarin.

S1 (F78) has putative S1 protein activity in SI and shared 55% amino acid homology with the SI S1 family protein from *Arabidopsis thaliana* (NP 196221) (Table S2). In *Papaver rhoeas*, the S1 protein exhibits specific pollen inhibitory activity *in vitro*. Pollen carrying the S1 allele is inhibited while pollen not carrying S1 is not inhibited [39]. However, S1 has not yet been documented in *Citrus*. In the present study, S1 (F78) showed obvious up-regulated expression in pollen (Figure 4) and in pistils 3 d after self-pollination of “Wuzishatangju” (Figure 5) which was similar to the expression pattern of the *F-box* gene (Figure 5). These results suggest that S1 (F78) may be a SI S1 family protein member involved in the pollen inhibitory activity of “Wuzishatangju” mandarin.

The general function of the ubiquitination pathway *i.e.*, ubiquitin-activating enzyme E1 (UBE1), UBE2 and UBE3 is to mediate protein degradation by the ubiquitin/26S proteosome pathway [40]. UBE1 first activates ubiquitin by covalently attaching the molecule to its active site cysteine residue, then UBE2 serves as an ubiquitin-carrier enzyme and performs the second step in the ubiquitination reaction; finally, UBE3 binds the target protein substrate and transfers ubiquitin from E2 cysteine to a lysine residue on the target protein [41]. The arm repeat-containing 1 (ARC1) and the skp1-cull-F-box (SCF) proteins, belonging to the UBE3 family, are involved in SSI and GSI responses, respectively [40]. ARC1 plays a positive role during the SI response. Antisense suppression of ARC1 in SI W1 plants of *Brassica napus* resulted in a partial breakdown of SI, demonstrating that these ARC1 antisense W1 plants had a functional *S*-locus protein, 11/*S*-locus Cys-rich (*SP11*/*SCR*) [42]. Qiao *et al.*[43] found that *AhSLF-S_2_* physically interacts with *S-RNases* in a non-allele–specific fashion probably through a proposed SCF^AhSLF−S2^ (Skp1/Cull or CDC53/F-box) complex that targets *S-RNase* destruction during a compatible rather than an incompatible response in *Antirrhinum hispanicum*. In our study, UBE2 (H858) and UBE3 (H556) were obtained from the F library (Table 1). Higher expression levels of UBE3 (H556) were detected in pollen of “Wuzishatangju” (Figure 4), indicating a specific function to regulate the pollen SI response through the ubiquitin/26S proteosome pathway. Compared to other candidate genes, UBE2 (H858) showed down-regulated expression in pollen (Figure 4), suggesting that they may be involved in the ubiquitination pathway in the pollen of “Wuzishatangju” mandarin.

Ca^2+^ can regulate pollen tube growth in the GSI system of *Papaver rhoeas* and the inhibition of pollen tube growth is correlated with elevated free calcium levels in the incompatible pollen tubes [44]. Ca^2+^-dependent protein kinases could lead to *S-RNase* phosphorylation [45] and pollen-tube polarity growth [46] in the GSI system of *Nicotiana alata*. In the SSI system of *Brassica*, Ca^2+^-binding protein is predominantly expressed in pistils and anthers and plays an important role in pollen-pistil recognition, pollen tube growth, and fertilization [47]. *OsPBP1* (*Oryza sativa* C2-domanin phospholipid-binding protein) has calcium concentration-dependent phospholipid-binding activity and is required for pollen fertility likely by regulating Ca^2+^ and phospholipid signaling pathways [48]. In this study, higher expression levels of CPBP (H115) were noted in pollen and stigmas of “Wuzishatangju” (Figure 3) as well as 2 d after self-pollination of “Wuzishatangju” pistils (Figure 4), which was consistent with the expression pattern of *OsPBP1* in *Oryza sativa*[48], implying that the CPBP are likely to act as a Ca^2+^ and phospholipid signaling factor to inhibit pollen tube growth and pollen fertilization of “Wuzishatangju” mandarin.

PCP putatively has a self-pollen rejection function and interacts with the male determining *S* specificity. It is essential for the rejection of incompatible pollen grains in the SI response of *Brassica* species [49]. *SP11/SCR* (*S*-locus protein 11 or *S*-locus Cys-rich) is the sole male determinant of SI in the *Brassica* genus and the *S_8_*-SP11 protein product of *SP11* transgenic plants was present in the tapetum, pollen, and pollen of late developmental stages [50]. Currently, 22 alleles of *SP11*/*SCR* have been identified and all of them encode proteins with similar characteristics to those of predicted pollen coat protein (PCP) family proteins (small, basic Cys-rich proteins). In our study, the highest expression level of PCP (H102) was detected in mature pollen of “Wuzishatangju” (Figure 3) and 2 d after self-pollination in the pistil of “Wuzishatangju” (Figure 4). In our previous study, we found that pollen tube growth was blocked at 3 d after self-pollination in “Wuzishatangju” [24]. These results suggest that PCP is likely to act as a male determining factor to inhibit self-pollen tube growth in “Wuzishatangju” mandarin.

Four candidate SI genes *i.e.*, RNase HII (F465), SDPP (F960), CWI (F1019), and SMP (F966) were also obtained (Table 1). RNase HII belongs to the RNases H type 2 family and shows putative RNase activity to degrade the RNA moiety of RNA-DNA hybrids [51]. SDPP putatively transcribes, specifically during sexual development, to control sexual reproduction of female and male flowers in dioecious plants [52]. CWI represents a putative function in sucrose partitioning between source and sink organs which could result in a block during the early stages of pollen development in tobacco [53]. SMP, encoding a putative group 1 late embryogenesis abundant (LEA) protein, is involved in the synthesis of late-maturing soybean seeds [54]. However, the relationship between these genes and the occurrence of the SI reaction is unclear. In this study, RNase HII, SDPP, and CWI were up-regulated in “Wuzishatangju” pollen (Figure 3). The highest expression level of SMP (F966) was observed 6 d after self-pollination in pistils of “Wuzishatangju” (Figure 4).

A high percentage of undescribed sequences (54.1%) were also obtained from the SSH (Table S1). This circumstance can account for the relatively high percentage of undescribed sequences encountered. Another reason to explain this high percentage may be that some ESTs could correspond to 3′ or 5′ untranslated regions (UTRs) which make it impossible to find homologues in available databases. However, most of these undescribed sequences can be classified as novel genes related to the SI reaction because only a few homologous genes related to SI have been identified.

## 4. Experimental Section

### 4.1. Plant Materials

Flower buds (1.0 cm × 0.5 cm) were collected from six-year-old trees of “Wuzishatangju” (six trees) and “Shatangju” mandarins (four trees) in an orchard of South China Agricultural University. Buds, leaves, petals, filaments, stigmas, styles, ovaries, pistils, and anthers were collected separately using tweezers and frozen immediately in liquid nitrogen and stored at −80 °C until later analysis. Mature pollen was used to construct SSH cDNA libraries, while the other organs were used for expression analyses of SI-related genes. Pistils of 0 h and 4 h, and 1 d, 2 d, 3 d, 4 d, 5 d, 6 d and 7 d after artificial self-pollination of “Wuzishatangju” and cross-pollination of “Wuzishatangju” × “Shatangju” were collected, immediately frozen in liquid nitrogen and stored at −80 °C for expression analysis.

### 4.2. Total RNA Extraction and mRNA Purification

Total RNA was extracted from mature pollen of “Wuzishatangju” and “Shatangju” according to Luo *et al.*[55] and pretreated with RNase-free DNase I (TaKaRa, Dalian, China). The quality and concentration of RNA were examined by ethidium bromide (EB)-stained 1.2% (*w*/*v*) agarose gel electrophoresis and spectrophotometry (Bio-RAD, Hercules, CA, USA). Two μg of mRNA were purified from total RNA using a PolyAttract^®^ mRNA Isolation System Kit III (Promega, Madison, WI, USA).

### 4.3. Construction of SSH Libraries

First-strand cDNAs were synthesized from about 2.0 μg of mRNA sample using AMV reverse transcriptase. Second-strand cDNAs were generated using T_4_ DNA polymerase, then digested with *Rsa* I, and ligated to different adaptors. Forward subtraction was carried out using cDNA from mature pollen of “Wuzishatangju” as the “tester” and cDNA from mature pollen of “Shatangju” as the “driver”. Reverse subtraction was performed using cDNA from mature pollen of “Shatangju” mandarin as the “tester” and cDNA from mature pollen of “Wuzishatangju” as the “driver” according to the manufacturer’s instructions (Clontech PCR-Select™ cDNA Subtractive Kit; TaKaRa, Dalian, China) following the procedure of Miao *et al.*[32].

### 4.4. Screening of SSH Libraries Using Colony-PCR and Reverse Northern Analysis

Inserted fragments were screened by colony-PCR using M13 primers (M13-F: 5′-GAGCGGATAAC AATTTCACACAGG-3′; M13-R: 5′-CGCCAGGGTTTTCCCAGTCACGAC-3′) (TaKaRa, Dalian, China). PCR was performed in a 25.0 μL reaction mixture containing 1× PCR buffer, 0.1 mM dNTPs, 0.4 μM M13 primers, and 1.0 U r*Taq* DNA polymerase (TaKaRa, Dalian, China). The PCR parameters were: 94 °C for 4 min then 35 cycles of 94 °C for 40 s, 55 °C for 40 s and 72 °C for 1.5 min, with a final 72 °C for 10 min. The quality and concentration of colony-PCR products were examined by EB-stained 1.0% (*w*/*v*) agarose gel electrophoresis and spectrophotometry (Bio-RAD, Hercules, CA, USA).

Reverse northern analysis was further carried out to screen up-regulated clones using the method of Miao *et al.*[32].

### 4.5. Bioinformatics Analysis of Expressed Sequence Tags (ESTs)

The nucleotide sequences of individual clones that exhibited up-regulated expression in the F and R libraries were sequenced according to the hybridization signal of reverse northern screening. All vector sequences were removed using VecScreen software in the National Center for Biotechnology Information (NCBI). All contigs and singlets were annotated according to the GO classification and the hierarchical structure using the Blast2GO suite. The Blast2Go program, which assigns the GO terms based on the BLAST definitions, was applied with an *E*-value < 10^−3^. If a transcript was annotated with more than one GO category, it was split equally among them. Ten ESTs from the F library and 15 ESTs from the R library were registered in GenBank (Table 1).

### 4.6. Expression Analysis of SI-Related Genes by SqRT-PCR and qPCR

Total RNA was extracted from various organs using the plant RNAout Kit (TIANDZ, Beijing, China). First-strand cDNA was synthesized in a 20.0 μL reaction mixture using 1.0 μg of total RNA from each sample by an Oligo(dT)_18_ primer and a Reverse Transcriptase M-MLV Kit according to the manufacturer’s instructions (TaKaRa, Dalian, China).

Specific primer pairs were designed for all SI candidate genes from the F and R libraries (Table S5) using Primer 5.0 software. The expression patterns of all candidate genes were first studied by SqRT-PCR using a pair of primers (Actin-F: 5′-CACACTGGAGTGATGGTTGG-3′ and Actin-R: 5′-ATTGGCCTTGGGGTTAAGAG-3′) as an internal control to normalize samples. Expression levels of candidate genes were quantified by qPCR in an iQ5 real-time PCR detection system (Bio-Rad, Hercules, CA, USA) using the SYBR *Ex*Script RT-PCR Kit (TaKaRa, Dalian, China). A sample of 25.0 μL of the qPCR reaction volume contained 12.5 μL SYBR^®^ Premix Ex*Taq*™ (TaKaRa, Dalian, China), 1.0 μL of each forward and reverse primers (5.0 μM), 8.5 μL ddH_2_O, and 2.0 μL cDNA (40 ng). Actin-F and Actin-R primers were used to amplify the citrus *actin* gene (accession No. GU911361) as a loading control to normalize samples in separate tubes. Controls (*actin* gene) and all SI candidate genes were run in triplicate and repeated twice (technical replicates). Relative expression levels of these genes were calculated using the 2^−ΔΔCT^ method [56]. Data were analyzed using iQ5 software in an iQ5 real-time PCR detection system (Bio-Rad, Hercules, CA, USA).

## 5. Conclusion

SSH approach is a useful tool to identify differently expressed genes in pollen from SI “Wuzishatangju” and SC “Shatangju” mandarins. Six candidate genes *i.e.*, an *F-box* gene (H304), UBE2 (H858), UBE3 (H556), S1 (F78), CPBP (H115), and PCP (H102) were obtained and their temporal and spatial expression characteristics were explored. Up-regulated expression of the six genes in pollen and in pistils at 3 d after self-pollination of “Wuzishatangju” shows that they are possibly involved in the SI reaction of “Wuzishatangju” mandarin. Further research will be necessary to elucidate why the different expression levels of these genes could result in the SI reaction of “Wuzishatangju”.

## Figures and Tables

**Table 1:** All potential pollen self-incompatibility (SI)-related genes obtained from the F and R libraries.

Seq. Name	Seq. Description	GenBank accession No.	Length (bp)	*E*-value	Similarity	Annotation
F library						

H102	Pollen coat-like protein (PCP)	JK724775	488	2.23 × 10^−31^	86.55%	Pollen development

H115	Calcium-dependent phospholipid binding protein (CPBP)	JK724776	805	3.67 × 10^−112^	86.40%	Calcium ion binding

H128	Pollen allergen Pla o1	JK724777	526	1.80 × 10^−35^	57.85%	Extracellular space
H264	Senescence-associated protein	JK724778	252	1.07 × 10^−36^	89.95%	Biological process
H304	F-box protein (F-box)	JK724779	446	3.04 × 10^−23^	87.80%	Kinase activity

H428	Actin depolymerizing factor (ADF)	JK724780	612	6.19 × 10^−85^	93.75%	Cytoskeleton
H543	Zinc finger protein	JK724781	372	4.58 × 10^−33^	69.80%	Transcription factor activity
H556	Ubiquitin-protein ligase E3 (UBE3)	JK724782	471	1.44 × 10^−62^	95.00%	CUL4 RING ubiquitin ligase
H858	Ubiquitin-conjugating enzyme E2 (UBE2)	JK724783	314	1.58 × 10^−35^	97.63%	ATP binding
H1092	Vacuolar H^+^-translocating inorganic pyrophosphatase	JK724784	808	1.33 × 10^−5^	64.56%	Inorganic diphosphatase activity

R library						

F15	Phospholipase	JK724785	248	7.28 × 10^−39^	77.30%	Serine/threonine kinase activity
F65	F-box and wd40 domain protein	JK724786	339	6.79 × 10^−52^	82.15%	G-protein complex

F78	Self-incompatibility S1 family protein (S1)	JK724787	252	1.38 × 10^−9^	62.83%	Pollen-pistil interaction

F960	Sexual differentiation process protein isp4-like (SDPP)	JK724788	840	7.23 × 10^−67^	83.80%	Transporter activity

F966	Seed maturation protein PM37 (SMP)	JK724789	246	5.47 × 10^−26^	87.90%	Biological process

F1166	Ca^2+^-dependent membrane-binding protein annexin	JK724790	212	1.25 × 10^−16^	7.50%	Calcium-transporting ATPase activity

F1166	Ca^2+^-dependent membrane-binding protein annexin	JK724790	212	1.25 × 10^−16^	87.50%	Calcium-transporting ATPase activity

F250	Alpha glucosidase	JK724791	478	9.75 × 10^−50^	83.65%	Glucosidase activity

F465	Ribonuclease HII family protein (RNaseHII)	JK724792	344	6.15 × 10^−18^	77.24%	Ribonuclease H activity

F499	Anther-specific protein LAT52 precursor	JK724793	181	1.67 × 10^−6^	70.80%	Extracellular space

F519	Actin 3	JK724794	692	4.47 × 10^−134^	99.50%	Cytoskeleton

F546	Pollen allergen Che a 1 precursor	JK724795	260	1.67 × 10^−14^	64.50%	Extracellular space

F1019	Cell wall invertase (CWI)	JK724796	420	9.75 × 10^−50^	83.65%	Hydrolase activity

F580	Zinc finger (C2H2 type) family protein	JK724797	373	3.65 × 10^−22^	75.20%	Transcription factor activity

F685	Auxin-repressed protein	JK724798	595	1.18 × 10^−9^	61.67%	Signal transduction

F822	WD-40 repeat protein-like (WD-40)	JK724799	465	6.61 × 10^−41^	94.25%	G-protein complex
